# Recent advances in understanding and managing retinal vein occlusions

**DOI:** 10.12688/f1000research.12886.1

**Published:** 2018-04-16

**Authors:** Daniel D. Esmaili, David S. Boyer

**Affiliations:** 1 Retina-Vitreous Associates Medical Group, 001 Wilshire Boulevard, Suite 301, Beverly Hills, CA 90211, USA; 2 Keck School of Medicine, University of Southern California, 1975 Zonal Avenue, Los Angeles, CA 90033, USA

**Keywords:** retina, vein occlusions, retinal vascular disease, anti-VEGF

## Abstract

Retinal vein occlusions are the second most common form of retinal vascular disease. Previously, laser treatment for branch retinal vein occlusion and intravitreal triamcinolone acetonide for central retinal vein occlusion were the standard of care. Recent studies have demonstrated that anti-vascular endothelial growth factor (anti-VEGF) agents have a superior safety and efficacy profile for the treatment of both branch and central retinal vein occlusions. The use of wide-field fluorescein angiography has also allowed better visualization of the retinal periphery. Despite the better documentation of retinal non-perfusion, laser photocoagulation to the areas of non-perfusion does not seem to result in a reduction of macular edema or reduction in treatment burden and has been relegated to patients who develop rubeosis or neovascularization of the retina. More recently, several studies have demonstrated the use of a long-acting dexamethasone implant administered intravitreally or triamcinolone administered in the suprachoroidal space as a viable approach to treat retinal vein occlusion.

## Introduction

Retinal vascular occlusions are the second most common form of retinal vascular disease after diabetic retinopathy ^1^. There are two major anatomic forms of retinal vascular occlusions: branch retinal vein occlusion (BRVO) and central retinal vein occlusion (CRVO). Furthermore, retinal vein occlusions (RVOs) can be classified as ischemic and non-ischemic occlusions, depending on the degree of non-perfusion based on the fluorescein angiogram ^2^. Wide-field angiography has become more available and is an enhanced method of determining non-perfusion.

BRVO is three to four times more common than CRVO and often occurs at the crossing of an artery and a vein. CRVO has a poorer prognosis than does BRVO, and ischemic CRVO has a poorer prognosis than does perfused CRVO. CRVO usually occurs from a thrombus in the central retinal vein at the level of the lamina cribrosa in the optic nerve ^3^.

## Randomized clinical studies

### SCORE study

A National Eye Institute-sponsored SCORE (Standard Care versus Corticosteroid for Retinal Vein Occlusion) study compared 1 and 4 mg of preservative-free intravitreal triamcinolone acetonide versus observation in patients who had both BRVO and CRVO ^4, 5^. BRVO treated in the SCORE study with 1 or 4 mg of triamcinolone versus focal laser showed that all groups gained three lines of vision in a similar fashion. CRVO treated in the SCORE study showed three lines of improvement with both 1 mg (21%) and 4 mg (26%) triamcinolone acetonide versus 7% with observation. The complication rates of cataracts and increased intraocular pressure were higher in the 4 mg triamcinolone group versus the 1 mg group. Despite the results of the SCORE study, intravitreal anti-vascular endothelial growth factor (anti-VEGF) therapy has replaced corticosteroids as the treatment of choice for patients with CRVO because of a more favorable side effect profile.

### Geneva study

The dexamethasone implant (DEX implant; Ozurdex, Allergan, Irvine, CA, USA) is a biodegradable form of micronized dexamethasone that releases 700 mg of dexamethasone over several months and is inserted in the office through a 23-gauge needle puncture. Two multi-center trials included patients with CRVO and BRVO ^6, 7^. At 180 days, 41% and 40% of eyes receiving 0.7 or 0.35 mg of dexamethasone, respectively, improved 15 letters versus 23% of sham.

Although there have not been head-to-head studies comparing all three anti-VEGF medications, the SCORE2 study recently investigated six monthly injections of bevacizumab or aflibercept and found no significant difference in the visual result when both were administered monthly for six months ^8^. An important learning point from this study was that frequent injections and early treatment led to better visual acuity results with all anti-VEGFs.

The SCORE2 study confirmed the MARVEL study ^9^, a six-month study that randomly assigned patients with macular edema due to BRVO to 0.5 mg of ranibizumab or 1.25 mg of bevacizumab. At six months, the mean gains in visual acuity were 18.1 letters for the ranibizumab group and 15.6 letters for the bevacizumab group.

Laser photocoagulation therapy was previously considered a treatment of choice for macular edema secondary to BRVO ^10^. Several studies have examined retinal photocoagulation to the areas of peripheral non-perfusion in eyes with CRVO and macular edema and showed no improvement in macular edema ^11^.

In the RELATE trial, subjects received 0.5 or 2 mg of ranibizumab for six months and then were randomly assigned to ranibizumab with laser photocoagulation or ranibizumab only. There was no long-term benefit in visual acuity, macular edema, or number of injections needed by the addition of laser treatment to ranibizumab ^11^.

Panretinal photocoagulation therapy (PRP) is still being used for the treatment of neovascular complications of CRVO such as neovascularization of the retina, iris, or angle. Owing to the emergence of anti-VEGF therapy, PRP is being used less frequently.

### Surgical treatments

Several surgical treatments have been attempted, but none has undergone strict scrutiny in randomized clinical trials. In addition, most studies were done before the availability of anti-VEGF drugs.

***Radial optic neurotomy.*** In this approach, an incision is made in the optic nerve and adjacent retina with a microvitreoretinal (MVR) blade during pars plana vitrectomy ^12^. The original idea was to reduce the congestion of the optic nerve by opening the scleral canal, although it has also been hypothesized that this procedure may allow the formation of a retinal-choroidal anastomosis. There have been mixed results of its safety and efficacy ^13^, and this procedure has fallen out of favor and is rarely performed.

***Surgical formation.*** Surgical formation of retinal-choroidal anastomosis, either by laser ^14^ or directly at the time of vitrectomy, has also been advocated for perfused CRVO and has shown favorable results in some patients. The potential complications, however, include vitreous hemorrhage and neovascularization. No long-term follow-up or randomized clinical trials have been performed.

***Vitrectomy.*** Vitrectomy with internal limiting membrane and panretinal endophotocoagulation for macular edema secondary to CRVO has not shown any improvement ^15, 16^.

***Arteriovenous sheathotomy.*** Arteriovenous sheathotomy for BRVO has been advocated by some to release the arteriovenous adhesion and decompress the vein at an arteriovenous crossing point ^17, 18^. Hypertension and atherosclerotic changes to the arteriole may impinge the vein and lead to thrombus formation. The surgery involves performing a vitrectomy and then using either scissors or a bent MVR blade to cut the sheath at an arteriovenous crossing point until the artery becomes mobile. If done early before there is complete sclerosis of the vein, the procedure has shown an improvement of visual acuity in some patients ^19, 20^. Arteriovenous sheathotomy has shown functional and anatomic outcomes similar to those of intravitreal triamcinolone in a comparative trial ^21^.

***Tissue plasminogen activator.*** Tissue plasminogen activator (tPA) has also been used, both intravitreally as well as through direct injection into a cannulated retinal vein ^22– 25^. This approach has not been compared with medical management in a comparative trial.

### Medical therapy

Case reports of patients with RVO treated with anti-thrombotic or thrombolytic medications, including clopidogrel, tPA (both intravitreal and into a vein), heparin, aspirin, low-molecular-weight heparin ^26^, or hemodilation ^27^, have shown variable results, not allowing a recommendation. Unfortunately, no significant improvements have been found. **

## Associated systemic findings and risk factors

Several systemic conditions have been associated with retinal vascular occlusions, including hyperviscosity and hypercoagulability conditions: specifically, protein C and protein S deficiency, prothrombin gene mutation, anti-thrombin abnormalities, anti-phospholipid syndrome (anti-cardiolipin and lupus anti-coagulant), factor V Leiden deficiency, and hyperhomocysteinemia ^28– 31^.

Hypertension, diabetes mellitus, renal disease, atherosclerosis, glaucoma ^32^, and blood lipid disorders have been identified as risk factors ^33– 36^. In a meta-analysis, however, only hyperhomocysteinemia and anti-cardiolipin antibodies were significantly associated with RVOs. With RVO and no other history of vascular occlusions, it is rare to find a coagulation defect. In younger patients (<50 years old), a work-up for underlying hypercoagulable disease should be considered.

Recently, a meta-analysis of patients with both BRVO and CRVO showed an increased risk of stroke, especially in subjects between 50 and 69 years of age ^37^. Similarly, a meta-analysis association was found for an increased risk of acute myocardial infarction ^38^. It is prudent to recommend that patients with retinal vascular occlusions have their primary care providers evaluate and optimize their cardiovascular risk factors.

## Anti-VEGF therapy

Currently, there are three anti-VEGF drugs that are available to treat both CRVO and BRVO. Both ranibizumab and aflibercept are US Food and Drug Administration-approved, while bevacizumab is an off-label use of an anti-VEGF drug approved for the treatment of metastatic colon cancer ^39^. Anti-VEGF therapy is now the treatment of choice for retinal venous occlusive disease.

### Bevacizumab

Bevacizumab (Avastin, Genentech, South San Francisco, CA, USA) is the off-label use of the humanized monoclonal antibody that binds all forms of VEGF-A and has been approved only for the treatment of metastatic colon cancer. The drug is compounded in small aliquots and is used in the treatment of vein occlusions as well as diabetes and age-related macular degeneration ^40, 41^. Bevacizumab has been shown to be an effective off-label anti-VEGF for controlling macular edema associated with RVOs.

### Ranibizumab

Ranibizumab (Lucentis, Genentech) is a monoclonal Fab fragment which binds all forms of VEGF. Randomized phase III clinical trials using ranibizumab have been performed to assess the safety and efficacy of ranibizumab for the treatment of macular edema secondary to RVO.

Ranibizumab for the treatment of macular edema following BRVO has been studied in the Efficacy and Safety of Ranibizumab Injection in Patients with Macular Edema Secondary to Branch Retinal Vein Occlusion (BRAVO) trial. In this study, 55% and 61% of patients receiving 0.3 and 0.5 mg ranibizumab, respectively, experienced a three-line improvement in vision compared with 29% in the control group. Continued *pro re nata* (prn) treatment showed stabilization of vision, although laser treatment was added in almost 50% of patients ^42^.

Ranibizumab for the treatment of macular edema following CRVO has been studied in the Ranibizumab for the Treatment of Macular Edema after Central Retinal Vein Occlusion Study: Evaluation of Efficacy and Safety (CRUISE). A total of 392 patients with macular edema secondary to CRVO were randomized to 0.3 mg of ranibizumab, 0.5 mg of ranibizumab, or sham injection. At 6 months, 46% and 48% of patients in the 0.3 and 0.5 mg groups, respectively, showed three lines of improvement of vision versus 17% in the control group ^43^.

### Aflibercept

Aflibercept (VEGF Trap-Eye, Regeneron, Tarrytown, NY, USA) is a fusion protein with portions of VEGF receptor 1 and 2 bound by a fragment crystallizable (FC) portion. Intravitreal aflibercept binds to the isoform of human VEGF-A and placental growth factor (PIGF) with a higher affinity than does ranibizumab. The VIBRANT trial evaluated the efficacy of aflibercept over grid laser treatment in patients with BRVO and macular edema. A total of 57% of affected eyes treated with aflibercept gained three lines or more of vision. The study also showed that after monthly injections for six months, less frequent injections could still maintain vision ^44^.

The VEGF Trap-Eye: Investigation of Efficacy and Safety in Central Retinal Vein Occlusion (COPERNICUS) and the General Assessment Limiting Infiltration of Exudate in Central Retinal Vein Occlusion with VEGF Trap-Eye (GALILEO) studies both evaluated the use of aflibercept in the treatment of macular edema from CRVO. In both studies, over 50% of treated eyes compared with 12% of control eyes gained three lines of vision ^45, 46^.

## New advances in the treatment of RVO

There have been few recent advances in the treatment of BRVO. The sustained benefits of ranibizumab for 24 months in the BRIGHTER study confirmed visual superiority of ranibizumab or ranibizumab plus laser (14.8 letters) versus laser for six months and then ranibizumab as needed after (+6 letters) ^47^. Smaller studies have confirmed or demonstrated that delay in treatment is associated with decrease in visual improvement compared with prompt treatment.

Several studies have evaluated predictors of macular edema recurrence and visual acuity in patients with BRVO. Recurrence of macular edema has been shown to be associated with the degree of non-perfusion of the central 1 mm ETDRS (Early Treatment Diabetic Retinopathy Study) circle or with an initial central retinal thickness of more than 570 ^48^. Disorganization of the retinal inner layers is a predictor of subsequent visual acuity improvement or decline following the first three monthly injections in patients with macular edema ^49^.

RVO-associated macular edema may be refractory to treatment with an anti-VEGF agent. Risk factors for suboptimal response include older age, shorter occlusion distance from the optic nerve, longer pre-treatment duration, and larger areas of non-perfusion. A study of eyes with macular edema from RVO that were refractory to treatment with an anti-VEGF agent revealed that treatment with a long-acting dexamethasone implant showed a small improvement in both optical coherence tomography (OCT) and vision ^50^.

A small study investigated ranibizumab, aflibercept, or dexamethasone implant injections in patients with suboptimal response to bevacizumab. Patients were assessed every three months for a year. At month 12, vision improved in 59% of patients. There was no difference between each therapy, but the number of injections varied from 3.30 for dexamethasone to 6.50 for aflibercept and 8.27 for ranibizumab ^51^.

Optical coherence angiography has given us an opportunity to study the superficial and deep capillary plexus in patients with retinal vascular diseases. The degree of perifoveal capillary non-perfusion has been correlated with visual function ^52^. Other studies have suggested that the deep capillary plexus may be more severely affected than the superficial plexus ^53^. It has been shown that eyes with a reduction in vessel density of the deep plexus compared with the superficial plexus did not recover after a dexamethasone implant ^54^.

Combination of an anti-VEGF and a corticosteroid drug for the treatment of RVO has been advocated, but there is a paucity of long-term studies supporting this approach. A study by Singer *et al*. showed that combination therapy with an anti-VEGF agent and dexamethasone implant led to a mean re-injection interval of 135 ± 36.4 days for patients with macular edema secondary to CRVO and BRVO as well as improvements in visual acuity and central foveal thickness ^55^. Unfortunately, there was no control group. However, combination therapy remains a possibility for difficult-to-treat eyes.

Recent studies have shown that VEGF suppression reduces macular edema and prevents vessel closure by leukocytes ^56^. This finding supports the results of the aflibercept COPERNICUS and GALILEO studies showing that a delay in treatment results in a decrease in eventual visual improvement compared with early treatment.

The degree of retinal non-perfusion may be prognostic for the chance of developing new vessels. The odds of developing neovascularization go from 0% with less than one disc area (DA) of non-perfusion to an 80% risk with 75 to 150 DA of non-perfusion ^57^. The use of wide-field fluorescein angiography has allowed us to better determine capillary non-perfusion and the risk of developing neovascular complications.

## Future

There is ample hope that new anti-VEGF drugs will be coming to market in the near future.

### Conbercept

Conbercept (KH902) has already shown significant improvements in patients with RVO in China ^58^. Conbercept blocks all VEGF-A isoforms as well as VEGF-B, VEGF-C, and PIGF.

### Brolucizumab

Brolucizumab (Alcon, a division of Novartis, Fort Worth, TX, USA) has shown preliminary efficacy and safety and appears to be a strong anti-VEGF drug designed for the treatment of neovascular age-related macular degeneration. A vein occlusion trial for this drug will be initiated soon.

Nanoparticles, liposomes, and other drug delivery systems hopefully will allow less-frequent injections of anti-VEGF agents or corticosteroids. Special needles and devices to allow more predictable penetration of a retinal vein are being developed with the hope that they may be used to inject tPA and other agents directly into the area of occlusion.

### Clearside Biomedical Inc.

Clearside Biomedical Inc. (Alpharetta, GA, USA) has introduced a novel approach to the treatment of RVOs, using a combination of a suprachoroidal delivery system for the delivery of corticosteroids and intravitreal aflibercept, to improve vision and decrease the treatment burden. In the TANZANITE study, 46 treatment-naïve patients with RVO received intravitreal aflibercept alone or the combination of aflibercept and concomitant suprachoroidal delivery of triamcinolone acetonide ^59^. At three months, the combination arm showed an increase in visual acuity and improved OCT compared with the aflibercept-alone cohort. A total of 74% of the combination-treated group did not receive any additional treatment over nine months.

In conclusion, RVOs continue to be a commonly encountered retinal condition. With the advent of improved therapies, we now have the ability to treat the secondary complications of neovascularization and macular edema. Future advances will allow for more effective treatments while hopefully minimizing the treatment burden for our patients.
